# Ameliorative Effects of HT074-Inula and Paeonia Extract Mixture on Acute Reflux Esophagitis in Rats via Antioxidative Activity

**DOI:** 10.3390/antiox13080891

**Published:** 2024-07-23

**Authors:** Young-Sik Kim, Yeonjin Park, Yongbin Kim, Hyo-Eun Son, Jinhui Rhee, Chang-Won Pyun, Chanoh Park, Hocheol Kim

**Affiliations:** 1 Department of Herbology, College of Korean Medicine, Woosuk University, Jeonju 54986, Republic of Korea; yjbsik@gmail.com (Y.-S.K.); drag8070@hanmail.net (Y.K.); coco1869@naver.com (H.-E.S.); qa6411@naver.com (J.R.); 2 Department of Herbal Pharmacology, College of Korean Medicine, Graduate School, Kyung Hee University, 26 Kyungheedae-ro, Dongdaemun-gu, Seoul 02447, Republic of Korea; yeonjin517@gmail.com (Y.P.); rookie1320@khu.ac.kr (C.P.); 3 NEUMED R&BD Institute, NeuMed Inc., 88 Imun-ro, Dongdaemun-gu, Seoul 02440, Republic of Korea; fleagles09@gmail.com; 4 Department of Herbal Pharmacology, College of Korean Medicine, Kyung Hee University, 26 Kyungheedae-ro, Dongdaemun-gu, Seoul 02447, Republic of Korea

**Keywords:** antioxidative activity, gastroesophageal reflux disease, HT074, *Inula britannica*, *Paeonia lactiflora*

## Abstract

HT074, a multiherbal mixture containing extracts from *Inula britannica* flowers and *Paeonia lactiflora* roots, is used in Korean medicine for gastric disorders. This study investigated the protective mechanisms of HT074 against acute reflux esophagitis (RE) in rats. Nitric oxide (NO) production and mRNA expression of antioxidant-related genes (*Nrf2*, *HO-1*, *SOD*, *CAT*, and *GPx2*) were evaluated in LPS-induced RAW 264.7 cells. Gastroesophageal reflux (GER) was induced in rats, followed by HT074 (100, 300 mg/kg) or ranitidine (50 mg/kg) administration. Esophageal damage and histological changes were assessed. Gastric pH and protein expression levels of Nrf2, HO-1, SOD, CAT, and GPx-1/2 were measured. HT074 pretreatment reduced NO production and increased the expression of HO-1, CAT, and GPx2 in LPS-induced RAW 264.7 cells. In GER-induced rats, HT074 significantly decreased esophageal lesions and increased the expression of HO-1, SOD, GPx-1/2, and Nrf2. HT074 did not affect gastric pH. These findings suggest that HT074 protects against GER-induced esophagitis by inhibiting NO production and enhancing antioxidant activity. Therefore, HT074 could be a promising therapeutic agent for GER disease.

## 1. Introduction

Gastroesophageal reflux disease (GERD) is a disorder characterized by bothersome symptoms and complications resulting from the regurgitation of stomach contents [1]. The prevalence of GERD is on the rise worldwide, with estimated rates ranging from 18.1 to 27.8% in North America, 8.8 to 25.9% in Europe, and 2.5 to 4.8% in Eastern Asia [2]. While GERD is more commonly observed in Western countries, recent epidemiological studies indicate a growing incidence in Asian countries [3]. This upward trend in GERD prevalence is attributed to lifestyle and behavioral changes, including smoking, dietary patterns, and obesity [4].

The multifaceted origins of abnormal gastric reflux involve the presence of weakened antireflux barriers at the gastroesophageal junction, leading to injury and inflammation of the esophageal mucosa. This damage results from an imbalance between aggressive factors, such as refluxed gastric juice, and defensive factors, including esophageal clearance, mucosal resistance, and endogenous antioxidant defense systems [5,6,7,8]. Therefore, the development of new products focuses on inhibiting aggressive factors or enhancing defensive factors.

Conventional treatments for GERD primarily rely on acid inhibitors, specifically proton pump inhibitors (PPIs) and type 2 histamine receptor antagonists (H_2_ blockers), which aim to reduce gastric acidity [9]. However, their effectiveness in rapidly alleviating GERD symptoms is marred by observations of relapses, incomplete mucosal healing, and complications associated with long-term use. Recent studies have indicated a potential association between long-term acid inhibitor use and the development of mucosal degeneration, polyps, and osteoporosis [10,11]. Ranitidine was once the most prescribed medication on the market due to its excellent effect on acid secretion reduction and its relatively long duration of action. However, it was withdrawn from the market because it was found to contain high levels of N-nitrosodimethylamine, which can cause cancer [12]. These underline the need for alternative treatment approaches, such as relatively safe plant extracts, that provide long-term relief while minimizing potential adverse effects.

Despite the favorable outcomes of existing therapies and their relative risk–benefit balance, there persists a continuous pursuit for the development of novel treatments offering both effectiveness and safety. Ongoing research explores alternative approaches to enhance patient outcomes while minimizing potential risks. Gastric acid stimulates the epithelial cells of the esophagus, which leads to the generation of reactive oxygen species (ROS) and induces oxidative stress [13]. The inflammatory process mediated by oxygen-derived free radicals causes damage to the esophageal mucosa. Therefore, therapeutic agents that scavenge reactive oxygen species (ROS) can serve as novel approaches for the treatment of GERD [14,15].

Natural products obtained from plants and agro-industrial waste have gained importance for their multiple effects in the health area, such as antioxidant and anti-inflammatory effects [16]. Such natural products have also been applied for GERD treatment. Beeswax alcohols (D-002) and chlorogenic acid (CGA) have been shown to suppress gastric mucosal injury by exerting antioxidant and anti-inflammatory effects [17,18,19]. These products have been effective in reducing esophageal lesions and oxidative stress markers in acute esophagitis models [20,21]. Additionally, *Artemisia asiatica* extract (DA-9601), prescribed for gastritis patients in Asia, exhibits antioxidant and cytoprotective effects in experimental animal models of mucosal damage, such as ethanol-triggered gastritis [22] and esophageal mucosal damage [23,24].

HT074 is a combination of two widely consumed herbal ingredients: *Paeonia lactiflora* Pall. Roots and *Inula britannica* L. flowers. These herbs have a history of use in herbal medicine and food preparations [25,26]. The formulation of HT074 was developed with the intention of harnessing the protective properties of these herbs against gastric mucosal damage. Previous studies have shown that when orally administered, HT074 can mitigate gastric mucosal injury induced by various factors, such as HCl/EtOH, indomethacin, and water immersion-restraint stress. This effect is attributed to the ability of HT074 to enhance gastric wall mucus production and inhibit the formation of oxygen free radicals [27]. *I. britannica* L., one of the herbal components of HT074, is known to contain sesquiterpene lactones and flavonoids. This herb has been extensively studied for its various biological activities, including its antioxidant, anti-inflammatory, antitumor, and hepatoprotective properties [28,29]. The bioactive compounds present in *I. britannica* contribute to its pharmacological efficacy and make it a valuable component of HT074. Pretreatment of *I. britannica* extract administered to rats with HCl/EtOH-induced gastritis increased the amount of adherent mucus and enhanced antioxidant activity [30]. *P. lactiflora* Pall., the other herbal component of HT074, contains bioactive compounds such as phenolic compounds and glycosides, including paeoniflorin and paeonol. These compounds are known to contribute to the beneficial effects exhibited by *P. lactiflora*, which include hepatoprotective, anti-inflammatory, antioxidant, and antitumor properties [31]. The roots of *P. lactiflora* and its main active compound, paeoniflorin, have demonstrated protective effects on the gastric mucosa against HCl/EtOH-induced gastric ulcers in mice [32,33] (Table 1). Based on previous findings regarding the protective effects of both *I. britannica* and *P. lactiflora* on gastric mucosa, it was hypothesized that HT074, which contains these two herbal components, may also have a protective effect on the gastroesophageal mucosa.

The objective of this experiment was to determine the esophageal mucosal protective effect of HT074 and its possible mechanisms in gastroesophageal reflux (GER)-induced rats. In this study, we observed esophageal mucosal lesions and histological changes. To investigate the underlying mechanisms, we measured gastric pH as well as the levels of Nuclear factor erythroid 2-related factor 2 (Nrf2), Hemo oxygenase-1 (HO-1), glutathione peroxidase (GPx), and Superoxide dismutase (SOD) in GER-induced rats.

## 2. Materials and Methods

### 2.1. Reagents

Penicillin/streptomycin and fetal bovine serum (FBS) were obtained from Gibco (Waltham, MA, USA). Dulbecco’s modified Eagle medium (DMEM) was obtained from WELGENE (Gyeongsan, Republic of Korea). Lipopolysaccharide (LPS) and Bradford reagent (B6916) were obtained from Merck (Darmstadt, Germany). 3-(4,5-Dimethyl-2-thiazolyl)-2,5-diphenyltetrazolium Bromide (MTT) and ranitidine were purchased from TCI Chemicals (Japan). RNAiso Plus, PrimeScript^TM^ RT Master Mix, and TB Green^®^ FAST qPCR Mix were purchased from Takara Bio (Shiga, Japan). DEPC was purchased from BYLABS (Hanam, Republic of Korea). Laemmli’s sodium dodecyl sulfate (SDS)-Sample buffer and ECL reagent were purchased from GenDEPOT (Katy, USA). Nrf2 (1:100, sc-365949), GPx-1/2 (1:100, sc-133160), catalase (Cat) (1:1000, sc-271803), HO-1 (1:500, sc-390991), SOD (1:1000, sc-101523), and β-actin (1:1000, sc-47778) antibodies were purchased from Santa Cruz Biotechnology (Dallas, TX, USA). Secondary antibodies (1:2000) were purchased from Bioss Inc. (Woburn, MA, USA). Black silk (4-0) was purchased from Ailee (Busan, Republic of Korea). Isoflurane was purchased from Hana Pharm (Seoul, Republic of Korea). With external standards for HPLC quantitative analysis showing a purity of over 97.0%, paeoniflorin was purchased from Tokyo Chemical Industry (Tokyo, Japan), and 1-O-acetylbritannilactone was purchased from NatureStandard (Shanghai, China), respectively. HPLC-grade acetonitrile and methyl alcohol were acquired from Honeywell International (Charlotte, NC, USA), and HPLC-grade phosphoric acid was purchased from DUKSAN Science (Seoul, Republic of Korea).

### 2.2. Sample Preparation

Paeonia extract mixture HT074 was provided by Neumed Inc. The preparation method for the extract mixture followed the same procedure as the previous method, summarized as follows [27]:

Dried roots of *P. lactiflora* and dried flowers of *I. britannica* were bought from Sinochem Pharmaceutical Co., Ltd. (Nanjing, China). The Prof. Hocheol Kim Department of Herbal Pharmacology, College of Korean Medicine, Kyung Hee University, authenticated the plant materials. The following procedure was followed to make the HT074 extract mixture: The dried plants were individually extracted for 3 h at 100 °C with distilled water. To facilitate filtration, amylase was added to the *P. lactiflora* extract. It was later inactivated. The extracts were percolated, concentrated, and spray-dried with dextrin (20% for *P. lactiflora* and 10% for *I. britannica*). Powdered extracts of *I. britannica* and *P. lactiflora* were combined at a ratio of 53:47.

### 2.3. High-Performance Liquid Chromatography (HPLC) Analysis

The levels of two marker compounds, 1-O-acetylbritannilactone and paeoniflorin, were quantified by HPLC. The analysis was performed on the Agilent 1260 HPLC system (Agilent Technologies, Palo Alto, CA, USA) equipped with a G7129A autosampler using a Sunfire™ C_18_-column (5 μm; 250 × 4.6 mm; Waters, Milford, MA, USA), a G7155A diode array detector (DAD), and a G7111A quaternary pump. The mobile phase, which was introduced at a flow rate of 1.0 mL/min, was composed of 0.5% phosphoric acid (A) and acetonitrile (B). The gradient elution for detection was carried out with the following parameters: 0–15–40–45–50–55 min, 20–20–70–70–20–20% solvent B. The detection wavelengths for 1-*O*-acetylbritannilactone and paeoniflorin were 210 nm and 235 nm, respectively.

### 2.4. Cell Culture

RAW264.7 murine macrophages were maintained in a humidified atmosphere with 5% CO_2_ at 37 °C. The cell culture growth medium comprised DMEM (Dulbecco’s Modified Eagle Medium) obtained from WELGENE (Republic of Korea), streptomycin (100 μg/mL) from Gibco (Waltham, MA, USA), supplemented with 1% penicillin (100 units/mL), and 10% FBS (fetal bovine serum) from Gibco (Waltham, MA, USA). The LPS utilized in the experiments was purchased from Sigma (St. Louis, MO, USA).

### 2.5. Cell Viability Assay

To perform the cell viability assessment, RAW264.7 cells were seeded in a 96-well plate at a concentration of 2 × 10^4^ cells per well and incubated at 37 °C for 24 h. After 24 h of incubation at 37 °C, the RAW264.7 cells were subjected to pretreatment with HT074 at various concentrations (10, 50, 125, and 250 μg/mL) for 1 h. Following this, the cells were exposed to LPS at a concentration of 100 ng/mL and incubated for 24 h. The MTT (3-(4,5-Dimethylthiazol-2-yl)-2,5-Diphenyltetrazolium Bromide) stock solution (5 mg/mL) was prepared by dissolving 5 mg MTT in 1 mL of 1 X × phosphate-buffered saline (PBS), and then filtered using a 0.2 μm filter. After the incubation period, the medium in each well was carefully removed. Next, 100 μL of MTT (3-(4,5-Dimethylthiazol-2-yl)-2,5-Diphenyltetrazolium Bromide) solution with a concentration of 0.5 mg/mL was added to each well, and the plate was incubated under the same conditions for 3 h. Subsequently, the MTT solution was discarded, and 100 μL of dimethyl sulfoxide (DMSO) was added to dissolve the formazan crystals that formed in viable cells. The plate was gently mixed to ensure complete dissolution. The absorbance of the formazan solution was measured at 570 nm using a microplate spectrophotometer, such as the SpectraMax^®^ ABS Plus from Molecular Devices, San Jose, CA, USA. The optical density measurements of the formazan crystals in the control group, which served as a representation of 100% cell viability, were utilized as a baseline reference. The MTT assay was conducted in triplicate for each concentration of HT074. MTT powder was acquired from TCI (Japan).

Cell viability (%) = Sample OD/Control OD × 100

### 2.6. Determination of Nitric Oxide (NO) Production

RAW264.7 cells were plated at a density of 2 × 10^4^ cells per well in a 96-well plate and incubated at 37 °C for 24 h. The cells were then pretreated with HT074 at concentrations of 10, 50, 125, and 250 μg/mL for 1 h, followed by stimulation with LPS at a concentration of 100 ng/mL for 24 h. After the incubation period, the culture medium was collected, and an equivalent volume of the Griess reagent (Sigma, St. Louis, MO, USA) was added to 100 μL of the culture supernatant. The mixture was incubated for a specific period to allow for the reaction between nitric oxide and the Griess reagent. Subsequently, the absorbance of the mixture was measured at 540 nm using a microplate spectrophotometer (SpectraMax^®^ ABS plus, Molecular Devices, CA, USA). The absorption coefficient was calibrated using a sodium nitrite standard solution.

### 2.7. RNA Extraction and Real Time PCR Analyses

RAW264.7 cells (at a density of 4 × 10^5^ cells per well in a 6-well plate) were pretreated with HT074 (at concentrations of 10, 50, 125, and 250 μg/mL) for 1 h, followed by stimulation with LPS (at a concentration of 100 ng/mL) for 24 h. After washing the cells with cold PBS, they were treated with 300 μL of RNAiso Plus (Takara, Japan).

Total RNA was extracted using RNAiso Plus (Takara, Japan) following the manufacturer’s instructions. The purified total RNA was dissolved in DEPC-treated solution (BYLABS, Hanam, Republic of Korea), and the concentration was determined using a SpectraMax^®^ ABS plus. For reverse transcription, 500 ng quantities of total RNA were used as templates with the PrimeScript^TM^ RT Master Mix (Takara Bio, Kusatsu, Japan).

qPCR was performed using 50 ng of total RNA. The expression of each gene was quantified using the TB Green^®^ FAST qPCR Mix (Takara, Japan) according to the manufacturer’s instructions. The PCR primer sequences can be found in Table 2.

The qPCR calculation used the 2^−ΔΔCt^ method. First, ΔCt was calculated as the difference in the threshold cycle between the target and reference genes. Then, ΔΔCt was calculated as the difference in ΔCt between the target and control samples. The result is expressed as a fold change.

(1) $$∆Ct={Ct}_{target\;gene}-{Ct}_{reference\;gene}$$

(2) $$∆∆Ct=∆{Ct}_{sample}-∆{Ct}_{control}$$

(3) $$Fold\;Change=2^{-∆∆Ct}$$

### 2.8. Animal Management

Male Sprague-Dawley rats, at the age of seven weeks and weighing 180–200 g, were obtained from DBL (Eumseong, Republic of Korea). The animal experiments were conducted under standard laboratory conditions with a temperature of 23 ± 3 °C, a humidity of 55 ± 5%, and a 12 h light/dark cycle for seven days prior to the experiments. The rats were provided with ad libitum access to food and water. During the housing period, the animals’ health status, including food intake, behavior changes, and body weight, was monitored once daily. No adverse events were observed. All experimental procedures were approved by the Woosuk University Institutional Animal Care and Use Committee (Ethic no. WS2022-6).

### 2.9. RE Surgery Rat Models and Treatment

The rats were administered distilled water via oral gavage 1.5 h prior to surgery. A fasting period of 18 h was observed before conducting the experiments, during which the rats had ad libitum access to water. Following an adaptation period, 32 rats were randomly allocated into four groups, each consisting of 8 rats (n = 8). Group 1 served as the control group for esophageal inflammation and injury. In Group 2, the rats were pretreated with ranitidine (50 mg/kg) obtained from TCI (Japan), which served as the positive control. In Groups 3 and 4, the rats were pretreated with HT074 at doses of 100 and 300 mg/kg, respectively, for the HT074 treatment groups. All drug treatments were administered 1.5 h prior to the surgery. The rats were anesthetized with respiratory anesthesia for esophageal inflammation. During anesthesia, a median incision of approximately 2 cm was made to access the abdominal region of the rats. The fundus and pylorus were ligated using black silk (4-0) from Ailee (Republic of Korea) to induce RE, taking care not to damage the vagus nerve during ligation. The incision was promptly sutured, and the animals were kept in a postoperative recovery area before being transferred back to their cages. All rats were euthanized 4.5 h after the surgical procedure. Isoflurane gas (Ifran, Hana Pharm, Seoul, Republic of Korea) was used for anesthesia via a small animal sedation system (JD-C-107A, Jeung Do B&P, Seoul, Republic of Korea), and esophageal tissues were removed for analysis.

### 2.10. Determination of Gastric Acid pH

After euthanizing, the gastric cavity of each rat was rinsed with 1 mL of PBS at pH 7.4 using a 1000 μL micropipette. Subsequently, the pH of the collected gastric fluid was measured using a pH meter (Orion 420A, Thermo Fisher, Waltham, MA, USA).

### 2.11. Esophageal Mucosal Lesion Ratio

After euthanasia, each rat’s esophagus was longitudinally incised to expose the damaged tissue. The inner mucous layer was subsequently rinsed with PBS, and the remaining tissue was carefully spread out and placed on paper. Images of the tissue were captured using a D300s camera (Nikon, Tokyo, Japan) for further analysis. The acquired images were processed using Image J software (version 1.53K, NIH, Bethesda, MD, USA) to calculate the ratio of tissue lesion. To determine the gross mucosal lesion ratio, the width of the area with esophageal mucosal lesion (measured in mm^2^) was divided by the width of the total esophageal area (measured in mm^2^). The resulting value was then multiplied by 100 to express the gross mucosal lesion ratio as a percentage.

### 2.12. Esophageal Histological Analysis

For histological analysis, the middle segment of the opened esophagus was isolated and subsequently fixed in 4% paraformaldehyde. After fixation, the esophageal specimens were embedded in paraffin and sliced into sections measuring 5 μm in thickness. Hematoxylin and eosin (H&E) staining was performed on the sections. The stained slides were mounted using mounting media. Subsequently, the slides were examined under an optical microscope (Eclipse 80i, Nikon, Tokyo, Japan), and digital images were captured for further analysis.

### 2.13. Western Blot

Tissues stored at −80 °C were immersed in liquid nitrogen and then placed in ice-cold microfuge tubes. Using scissors, the tissues were cut into small pieces. Tissue samples were lysed in 300 μL of RIPA buffer (Biosesang, Yongin, Republic of Korea) containing protease inhibitor (GenDEPOT, Katy, TX, USA). The lysates were centrifuged at 4 °C and 13,000 rpm for 15 min, and the resulting supernatant was collected and stored at −80 °C until further analysis.

The protein concentrations were determined with the Bradford assay. After quantification of the protein concentration of each sample, 30 μg of proteins were mixed with Laemmli’s SDS-Sample buffer (GenDEPOT, Katy, TX, USA) at a 4:1 ratio and then subjected to heat at 95 °C for 5 min. The proteins were separated by 12% SDS-polyacrylamide gel electrophoresis (PAGE). Following electrophoresis, the proteins were transferred onto polyvinylidene difluoride membranes (PVDF, Thermo Fisher, Waltham, MA, USA). Non-specific binding was blocked by incubating the membranes with 5% non-fat dry milk in TBST buffer (Tris-buffered saline and 0.05% Tween 20) for 2 h at room temperature. The membranes were then incubated overnight at 4 °C with primary antibodies purchased from Santa Cruz: Nrf2 (1:100, sc-365949), GPx-1/2 (1:100, sc-133160), catalase (1:1000, sc-271803), HO-1 (1:500, sc-390991), SOD (1:1000, sc-101523), and β-actin (1:1000, sc-47778). Subsequently, the membranes were washed four times with TBST buffer for 10 min at room temperature and incubated with horseradish peroxidase-conjugated secondary antibodies (1:2000; Bioss Inc., Woburn, MA, USA) for 2 h at room temperature. After an additional four washes with TBST buffer for 10 min at room temperature, the protein bands were visualized using ECL reagent (GenDEPOT, Katy, TX, USA) according to the manufacturer’s instructions and captured using a Chemiluminescence imaging system (CAS-400SM, Davinch-K, Seoul, Republic of Korea). The relative optical densities of the protein bands were analyzed using ImageJ.

### 2.14. Statistical Analysis

Statistical analyses were performed using Prism 8 (GraphPad Software, Inc., La Jolla, CA, USA). Data were analyzed using one-way Analysis of variance (ANOVA) with Dunnett’s multiple comparison test when the normality of distribution (the Kolmogorov–Smirnov normality test) and homogeneity of variances (Bartlett’s) were confirmed. If the data were not normally distributed, we analyzed the data for statistical difference using the Kruskal–Wallis test with Dunn’s post hoc test. If there were heterogeneous variances in the data, the data were analyzed using Brown–Forsythe–Welch ANOVA with Dunnett’s T3 multiple comparison test. All data are presented as the mean ± standard deviation (SD). A *p* value < 0.05 was considered to indicate statistical significance throughout the study. The significance level is represented with asterisks (* *p* < 0.05, ** *p* < 0.01, *** *p* < 0.001, **** *p* < 0.0001; ns, non-significant).

## 3. Results

### 3.1. HPLC Analysis of HT074

**Figure 1:**
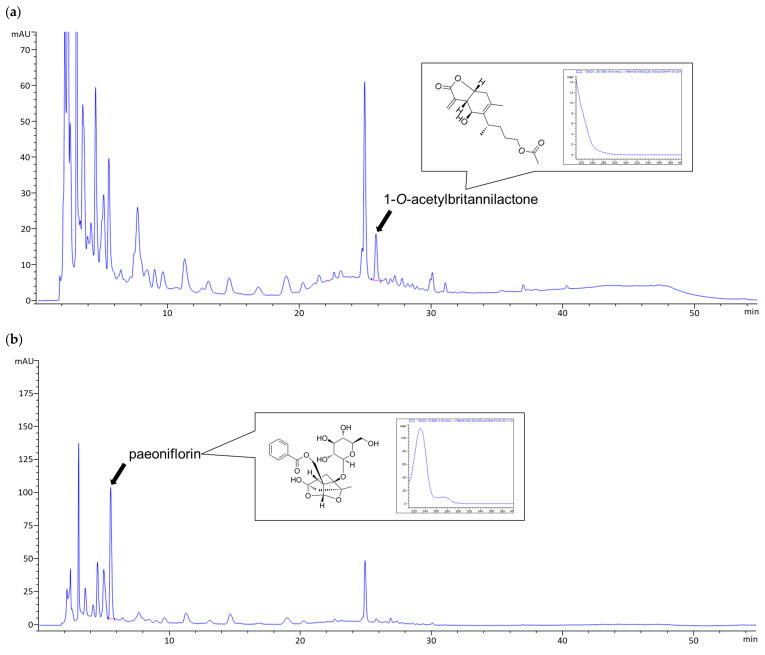
High-performance liquid chromatograms of HT074 extract at (**a**) 210 nm, (**b**) 235 nm. Arrows in (**a**,**b**) show the peaks of 1-*O*-acetylbritannilactone and paeoniflorin.

The HPLC chromatogram of HT074 is presented in Figure 1. The concentrations of 1-O-acetylbritannilactone and paeoniflorin were 2.38 mg/g and 23.78 mg/g, respectively, in Lot no. 220801503.

### 3.2. Effect of HT074 on Cell Viability in RAW264.7 Macrophages

**Figure 2:**
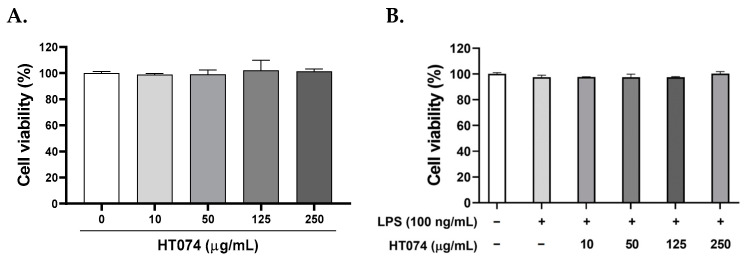
Effect of HT074 on the viability of LPS-treated and untreated RAW264.7 cells. (**A**,**B**) MTT assay was performed to evaluate the viability of RAW264.7 cells following treatment with or without 100 ng/mL LPS or 10–250 µg/mL HT074 for 24 h. Cell viability was determined using the MTT assay (n = 3).

To assess the effect of HT074 on cell viability in RAW264.7 cells, different concentrations (10, 50, 125, and 250 μg/mL) of HT074 were administered, followed by the MTT assay. The results showed that cell viability was not impacted by any of the tested concentrations of HT074 after 24 h of treatment (Figure 2A). Additionally, treatment with 100 ng/mL of LPS for 24 h did not affect the viability of RAW264.7 cells (Figure 2B). Based on these findings, we proceeded with experiments using various concentrations of HT074.

### 3.3. HT074 Attenuated LPS-Induced NO Production and Induced Antioxidant Gene Expression in RAW264.7 Macrophage Cells

**Figure 3:**
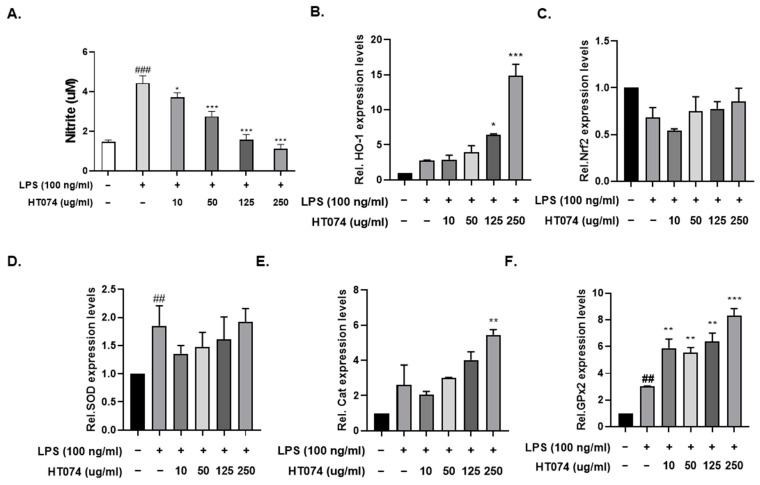
The effect of HT074 on NO synthesis and the expression of antioxidant markers in RAW 264.7 macrophages stimulated by LPS. RAW 264.7 cells were treated with HT074 at the designated concentrations (0, 10, 50, 125, and 250 μg/mL) with LPS (100 ng/mL) for 24 h. (**A**) The quantity of NO in the cell clarified solution was treated with Griess reagent to measure the amount of NO production. (**B**–**F**) The mRNA expression levels of antioxidant marker genes were measured by real-time PCR. The significance was determined by one-way ANOVA with Dunnett’s multiple comparison test. ## *p* < 0.01, ### *p* < 0.001 vs. control; * *p* < 0.05, ** *p* < 0.01, *** *p* < 0.001 vs. cells treated with LPS alone. Values are shown as the mean ± standard deviation.

We quantified LPS-triggered NO production in RAW264.7 cells using the Griess assay. We investigated the inhibitory effect of HT074 on LPS-triggered NO production by pretreating cells with HT074. Pretreated cells with HT074 exhibited a concentration-dependent attenuation of LPS-triggered NO production (Figure 3A). Furthermore, we examined the impact of HT074 on antioxidant gene expression in RAW264.7 cells. Treatment with HT074 significantly increased the expression levels of *HO-1*, *CAT*, and *GPx2* mRNA compared to LPS-treated RAW264.7 cells in a dose-dependent manner (Figure 3B,C,E,F). However, *Nrf2* and *SOD* mRNA expression levels did not significantly increase in the HT074-treated group compared to the LPS-treated group. Nevertheless, these expression levels exhibited a dose-dependent increase following HT074 treatment (Figure 3C,D). These findings indicate that HT074 inhibits LPS-induced NO production by increasing the expression of antioxidant-related enzymes in vitro.

### 3.4. Effects of HT074 on Esophageal Mucosal Lesion Ratio and pH of Gastric Contents Induced by Reflux Esophagitis (RE)

**Figure 4:**
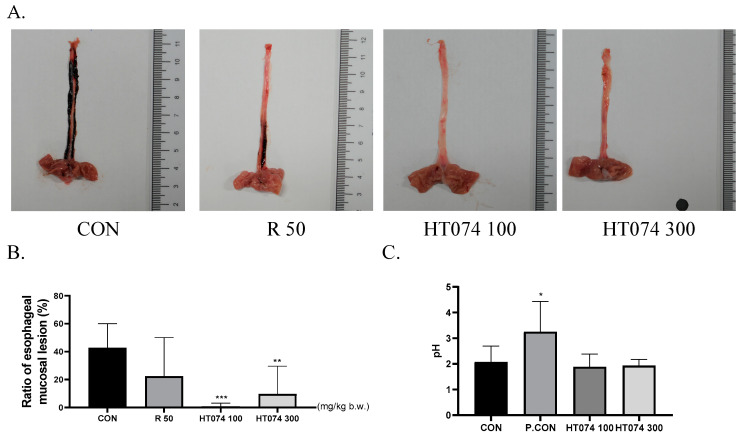
Protective effects of HT074 on RE in rats induced with gastric acid reflux. (**A**) Morphological examination of the esophagus in each group, (**B**) ratio of esophageal damage, (**C**) pH of gastric contents. Rats were administered orally with distilled water (CON), ranitidine 50 mg/kg (R 50), HT074 100 mg/kg (HT074 100), and HT074 300 mg/kg (HT074 300) 1.5 h before inducing acute reflux esophagitis. The significance was determined by one-way ANOVA with Dunnett’s multiple comparison test (ratio of esophageal damage) and the Kruskal–Wallis test with Dunn’s post hoc test (pH). * *p* < 0.05, ** *p* < 0.01, *** *p* < 0.001, vs. RE-controlled rats. Values are shown as the mean ± standard deviation.

To investigate the effectiveness of HT074 against RE, we surgically induced gastroesophageal reflux in rats. As shown in Figure 4A, the HT074-treated group exhibited a significant decrease in the esophageal mucosal lesion ratio compared to the RE control group. Notably, at concentrations of 100 and 300 mg/kg, the mucosal lesion ratios were comparable to those of the positive control group (ranitidine 50 mg/kg). In Figure 4B, wide dark red and long hemorrhagic lesions can be observed in the esophagus of the RE control group. However, the HT074-treated group demonstrated a significant reduction in esophageal hemorrhagic lesions compared to the control group. Particularly, at concentrations of HT074 100 and 300 mg/kg, the extent of the rat esophageal mucosal lesion was superior to that of the positive control group (ranitidine 50 mg/kg). HT074 had no effect on pH, whereas the ranitidine treatment group significantly increased gastric acid pH compared to the RE control group (Figure 4C).

### 3.5. Effects of HT074 on Histological Changes in Esophagus Triggered by RE

Histological observations revealed significant tissue damage in the esophageal tissue of the RE control group, characterized by mucosal damage, epithelial congestion, and mucosal and submucosal hemorrhage following acid reflux surgery. However, these changes were not observed in the HT074-treated group. Particularly, HT074 exhibited significant improvement compared to ranitidine.

**Figure 5:**
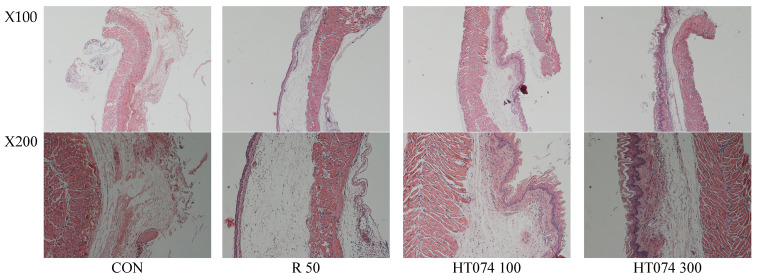
Effects of HT074 on histological changes in rats induced with esophageal reflux. Rats were administered orally with distilled water (CON), ranitidine 50 mg/kg (R 50), HT074 100 mg/kg (HT074 100), and HT074 300 mg/kg (HT074 300) 1.5 h before inducing acute reflux esophagitis.

Figure 5 confirms that the RE control group exhibited significant damage to the thickness of the epithelial cell layer and the lamina propria layer. In contrast, the esophageal tissue treated with HT074 and ranitidine showed evident protection against these lesions when compared to the RE control group.

### 3.6. HT074 Regulated Antioxidant Effect on Rat Esophageal Tissue

**Figure 6:**
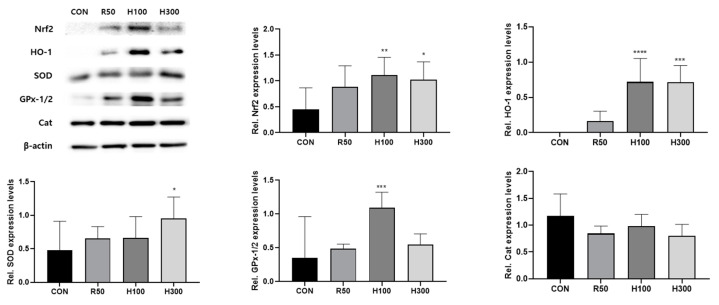
The effect of HT074 on the expression levels of antioxidation-related proteins in esophageal tissues of rats. Rats were administered orally with distilled water (CON), ranitidine 50 mg/kg (R50), HT074 100 mg/kg (H100), or HT074 300 mg/kg (H300) 1.5 h before acute reflux esophagitis was induced. Data are presented as the mean ± SD. The tissue lysates for each group were resolved by SDS–PAGE and immunoblotted with antioxidant markers. Densitometry of the Western blot bands’ antioxidant markers was performed using ImageJ, and ratios of target gene to β-actin were calculated. The significance was determined by one-way ANOVA with Dunnett’s multiple comparison test (Nrf2, SOD), Brown–Forsythe–Welch ANOVA with Dunnett’s T3 multiple comparison test (CAT), and the Kruskal–Wallis test with Dunn’s post hoc test (HO-1, GPx-1/2). * *p* < 0.05, ** *p* < 0.01, *** *p* < 0.001, **** *p* < 0.0001 vs. controlled rats.

We conducted experiments to verify the antioxidant effect on the tissues of the GERD model. The results demonstrated that HT074 treatment significantly increased the expression of HO-1 (*p* < 0.0001 in 100 mg/kg, *p* < 0.001 in 300 mg/kg), SOD (*p* < 0.05 in 300 mg/kg), GPx-1/2 (*p* < 0.001 in 100 mg/kg), and Nrf2 (*p* < 0.01 in 100 mg/kg, *p* < 0.05 in 300 mg/kg), consistent with the in vitro findings. However, no significant difference was observed in CAT expression (Figure 6).

## 4. Discussion

Pretreatment of the RAW 264.7 cells with HT074 attenuated LPS-induced NO production. Furthermore, HT074 treatment dose-dependently increased the expression levels of *HO-1, Nrf2, CAT*, and *GPx2* mRNA compared to LPS-treated RAW 264.7 cells. In rats, oral administration of HT074 effectively reduced the esophageal lesions induced by GER. HT074 did not have any significant impact on gastric pH levels. In tissues of the GERD model, HT074 treatment led to an increase in the expression of HO-1, SOD, GPx-1/2 and Nrf2, which aligns with the in vitro results. However, no significant differences were observed in the expression of CAT. These findings highlight the potential of HT074 as a therapeutic agent for managing GERD.

We performed experiments to determine the intracellular antioxidant effects of HT074. Pretreatment of LPS-induced RAW264.7 cells with HT074 reduced NO production in a concentration-dependent manner. The LPS-induced RAW 264.7 cell model is commonly used to screen anti-inflammatory and antioxidant activity [35,36]. The most significant indicator of cellular inflammation induced by LPS is the overproduction of inflammatory mediator NO [37]. It is well established that ROS can initiate the peroxidation of polyunsaturated fatty acids present in cellular membranes [38]. Excessive production of ROS and oxidative stress lead to the generation of high levels of NO as part of the inflammatory response [39]. These results suggest that the esophageal protective effects of HT074 might be mediated by the antioxidant effects.

HT074 reduced GER-induced esophageal lesions by 97.9% and 77.4% at doses of 100 and 300 mg/kg, respectively, compared to the control group in the rat model of GERD. The HT074 groups exhibited a higher inhibition rate compared to the ranitidine 20 mg/kg group (47.5%). The acute rat reflux esophagitis model is widely used in GERD studies. In this model, the pylorus is ligated to prevent gastric acid from entering the duodenum, and the forestomach is also ligated to reduce stomach volume, thus facilitating the reflux of gastric acid into the esophagus [40]. In this study, the esophageal tissue of the control group exhibited tissue injury and hemorrhage induced by the inflammatory response. As is commonly known, gastric acid reflux causes various forms of esophageal damage, including inflammation, ulceration, and disruption of the normal squamous epithelium [41]. The reduction of lesions in this model indicates its effectiveness in treating reflux esophagitis [42]. The results indicate that HT074 effectively mitigated esophageal mucosal damage in rats with acute esophagitis.

Given that symptoms commonly associated with GERD are typically linked to reflux episodes characterized by reduced pH levels, lower minimum pH values, and prolonged acid clearance times [41], an assessment of the gastric content pH levels was conducted. The administration of HT074 did not have an impact on pH levels, while ranitidine showed significant effects on the pH level of gastric contents, confirming its inhibitory effects on acid secretion. Although a previous study reported a decrease in acidity [27], it was not statistically significant in the current investigation. Thus, it appears that the esophageal protective effect of HT074 does not depend on gastric acid secretion. These properties of HT074 suggest that it can suppress GERD without causing various side effects, such as hypochlorhydria, rebound hypersecretion, and ventilator-associated pneumonia, commonly associated with the short-term or long-term use of acid secretion inhibitors [43,44].

Nrf2 is a crucial transcription factor involved in the regulation of antioxidant protein expression, which plays a protective role against oxidative damage [45]. The half-life of the Nrf2 protein is short under normal circumstances [46], which helps maintain cellular levels of Nrf2 at a low level to prevent transient activation of the antioxidant response. ROS induce changes in the Nrf2 complex and enhance the transcription of genes such as HO-1. Therefore, the Nrf2/HO-1 pathway can serve as a biomarker of oxidative stress response under pathological conditions [47]. Several studies have demonstrated the effective amelioration of inflammatory injury to the esophageal mucosa through activation of the Nrf2/HO-1 antioxidant pathway. In acute RE rats, extracts from Rhei Rhizoma, Gardeniae Fructus, and Curcumae Longae Rhizoma protected the esophageal mucosa by increasing the expression level of Nrf2 [24,47,48]. In the present study, HT074 increased the mRNA expression of *HO-1* in RAW264.7 cells, induced oxidative damage by LPS, and increased the protein expression levels of Nrf2 and HO-1 in rats. These results suggest that the antioxidant effects through the induction of HO-1 mediated by Nrf2 are involved in the esophageal protective effect of HT074.

Enzymes such as SOD, GPx, CAT, and others collectively participate in the metabolic pathway of free radicals, contributing to the elimination of free radicals [49]. Especially, SOD plays a role in regulating and expressing antioxidant enzymes in response to oxidative stress, while GPx functions to suppress oxidative stress and maintain the balance of oxidation and reduction [50,51]. If these enzymes are damaged due to oxidative stress, various degenerative disorders can occur. SOD swiftly converts superoxide (O_2_^−^) into hydrogen peroxide (H_2_O_2_), while GPx and CAT subsequently transform H_2_O_2_ into water (H_2_O), effectively eliminating oxidative stress [49]. The decreased antioxidant activity of SOD can contribute to esophageal damage in GERD patients and lead to cellular damage caused by free radicals. In other words, SOD can serve as a biomarker for the treatment of reflux esophagitis [52]. Previous studies have shown that HT074 protects the gastric mucosa by eliminating free radicals through a mechanism involving sulfhydryl compounds and promotes mucin secretion during gastrointestinal injury [27]. Other studies on various diseases have shown that *I. britannica* extract and its flavonoid fractions activate SOD and catalase, thereby exhibiting hepatoprotective effects in rats [53,54]. Additionally, several flavonoid compounds derived from *I. britannica* have been reported to increase CAT activity in rat cortical cells [55]. *P. lactiflora* extract has been found to enhance SOD, GPx, and CAT levels in the liver tissues of rats [56], and it has also been shown to prevent the loss of antioxidant enzyme activities, including SOD, GSH-Px, and CAT, in lung fibroblast MRC-5 cells [57]. Like previous studies, in this study, HT074 has been found to mitigate GER-induced lipid peroxidation and recover the reduction of antioxidant enzymes such as SOD and GPx-1/2. The antioxidant effect of HT074 was also confirmed through analysis of mRNA expression of antioxidant-related genes, such as *CAT* and *GPx2*, in RAW264.7 cells induced with oxidative damage by LPS. Based on these findings, our results suggest that the esophageal protective effect of HT074 is mediated by its antioxidant properties.

This study holds important implications for the potential treatment of GERD with natural substances, although there are limitations to the experiment. We used a model that induced gastroesophageal reflux over a short period and administered the extract only once. However, since GERD tends to worsen over a long period, further evaluation of the long-term effects of the extract using a long-term induction model is necessary. We are preparing for this and aim to confirm it through subsequent studies.

## 5. Conclusions

In summary, the results of this study revealed that oral administration of HT074 extract demonstrates a protective effect on the esophagus in rats with GER-induced esophagitis. The observed effect of HT074 is attributed to its ability to inhibit nitric oxide (NO) production and modulate antioxidant activity, leading to the activation of key cellular factors, including Nrf2, HO-1, GPx-1/2, SOD, and catalase. These findings support the potential of HT074 extract as a valuable therapeutic intervention for managing GERD.

## Figures and Tables

**Table 1:** Summary of previous research on the gastroprotective effects of *P. lactiflora* and *I. britannica*.

Research Objective/Test Model	Type of Study/ Species	Samples	Dose/Administration	Results/Mechanisms	Reference
Radical scavenging effects /DPPH, ABTS	In vitro	HT074	0–1000 μg/mL (DPPH) 20–20,000 μg/mL (ABTS)	DPPH, ABTS radial↓	Kim et al. (2018) [34]
PGE2 levels	In vitro/ AGS cell	HT074	50, 100, 200 μg/mL	PGE2 levels↑	Kim et al. (2019) [27]
Gastroprotection against HCl/EtOH	In vivo SD rats	HT074	30, 100, 300 mg/kg oral	Gastric lesions (100, 300 mg/kg)↓ Gastric wall mucus↑ mediated by endogenous sulfhydryl compounds	Kim et al. (2019) [27]
Gastroprotection against HCl/EtOH	In vivo SD rats	HT074	100, 300 mg/kg oral	Gastric lesions↓ Gastric SOD, GSH, CAT levels ↑ MUC5AC mRNA expression↑ Gastric wall mucus↑	Kim et al. (2018) [34]
Gastroprotection against HCl/EtOH	In vivo SD rats	Inulae Flos extract	100, 300 mg/kg, oral	Gastric lesions↓ SOD, GSH, CAT activities↑ MDA levels↓ PGE2 concentrations↑	Kim et al. (2020) [30]
Gastroprotection against HCl/EtOH	In vivo ICR mice	*P. lactiflora* extract	10, 100 mg/kg, oral	Gastric lesions↓ NO production↓	Bae et al. (2015) [32]
Gastroprotection against water immersion restraint stress	In vivo/ SD rats	HT074	30, 100, 300 mg/kg	Gastric lesions (300 mg/kg)↓	Kim et al. (2019) [27]
Gastroprotection against indomethacin	In vivo/ SD rats	HT074	30, 100, 300 mg/kg	Gastric lesions (300 mg/kg)↓	Kim et al. (2019) [27]
Gastric secretion /Pylorus-ligation	In vivo/ SD rats	HT074	100, 300 mg/kg	Gastric volume↓ Acidity↓ Total acidity↓	Kim et al. (2019) [27]

Note: ↑: Increased, ↓: Decreased.

**Table 2:** Sequences of the primers.

Genes	Forward Primer (5′–3′)	Reverse Primer (5′–3′)
*GAPDH*	GGCACAGTCAAGGCTGAGAATG	ATGGTGGTGAAGACGCCAGTA
*SOD*	TTCTGGACAAACCTGAGCCCTAA	GAACCTTGGACTCCCACAGACAC
*HO-1*	ATTTGTCCGAGGCCTTGAA	CCAGGGCCGTATAGATATGGTA
*CAT*	CAGATGTGAAGCGCTTCAACAG	GTTGGCAATGTTCTCACACAGG
*GPx2*	TGCCCTACCCTTATGACGAC	TCGATGTTGATGGTCTGGAA
*Nrf2*	ATTGCCACCGCCAGGACTAC	CCAAGATCTATGTCTTGCCTCCA

## Data Availability

The data presented in this study are available on request from the corresponding author.

## Abbreviations

ANOVA	Analysis of variance
CAT	Catalase
CGA	Chlorogenic acid
DAD	Diode array detector
DMEM	Dulbecco’s modified Eagle medium
DMSO	Dimethyl sulfoxide
GER	Gastroesophageal reflux
GERD	Gastroesophageal reflux disease
GPx	Glutathione peroxidase
H2 blockers	Type 2 histamine receptor antagonists
H&E	Hematoxylin and eosin
HPLC	High-performance liquid chromatography
LPS	Lipopolysaccharide
MTT	3-(4,5-Dimethylthiazol-2-yl)-2,5-Diphenyltetrazolium Bromide
NO	Nitric oxide
Nrf2	Nuclear factor erythroid 2-related factor 2
PBS	Phosphate-buffered saline
PPIs	Proton pump inhibitors
RE	Reflux esophagitis
ROS	Reactive oxygen species
SD	Standard deviation
SOD	Superoxide dismutase

## References

[1] Vakil N., van Zanten S.V., Kahrilas P., Dent J., Jones R., Global Consensus G. (2006). The Montreal definition and classification of gastroesophageal reflux disease: A global evidence-based consensus. Am. J. Gastroenterol..

[2] El-Serag H.B., Sweet S., Winchester C.C., Dent J. (2014). Update on the epidemiology of gastro-oesophageal reflux disease: A systematic review. Gut.

[3] Jung H.K. (2011). Epidemiology of gastroesophageal reflux disease in Asia: A systematic review. J. Neurogastroenterol. Motil..

[4] Fujiwara Y., Higuchi K., Watanabe Y., Shiba M., Watanabe T., Tominaga K., Oshitani N., Matsumoto T., Nishikawa H., Arakawa T. (2005). Prevalence of gastroesophageal reflux disease and gastroesophageal reflux disease symptoms in Japan. J. Gastroenterol. Hepatol..

[5] Kahrilas P.J., Lee T.J. (2005). Pathophysiology of gastroesophageal reflux disease. Thorac. Surg. Clin..

[6] Fox M., Forgacs I. (2006). Gastro-oesophageal reflux disease. BMJ.

[7] Tsuboi K., Hoshino M., Sundaram A., Yano F., Mittal S.K. (2012). Role of the lower esophageal sphincter on esophageal acid exposure—A review of over 2000 patients. Trop. Gastroenterol..

[8] Nelkine L., Vrolijk M.F., Drent M., Bast A. (2020). Role of antioxidants in the treatment of gastroesophageal reflux disease-associated idiopathic pulmonary fibrosis. Curr. Opin. Pulm. Med..

[9] Chiba N., De Gara C.J., Wilkinson J.M., Hunt R.H. (1997). Speed of healing and symptom relief in grade II to IV gastroesophageal reflux disease: A meta-analysis. Gastroenterology.

[10] Chen C.H., Lin C.L., Kao C.H. (2016). Gastroesophageal reflux disease with proton pump inhibitor use is associated with an increased risk of osteoporosis: A nationwide population-based analysis. Osteoporos. Int..

[11] Malfertheiner P., Kandulski A., Venerito M. (2017). Proton-pump inhibitors: Understanding the complications and risks. Nat. Rev. Gastroenterol. Hepatol..

[12] Perisetti A., Goyal H., Tharian B. (2021). The ‘burn’ of ranitidine recall: Current insights and mitigation strategies. Eur. J. Gastroenterol. Hepatol..

[13] Altomare A., Guarino M.P., Cocca S., Emerenziani S., Cicala M. (2013). Gastroesophageal reflux disease: Update on inflammation and symptom perception. World J. Gastroenterol..

[14] Wetscher G.J., Hinder R.A., Bagchi D., Hinder P.R., Bagchi M., Perdikis G., McGinn T. (1995). Reflux esophagitis in humans is mediated by oxygen-derived free radicals. Am. J. Surg..

[15] Deng Y., Pan L., Qian W. (2019). Associations between the severity of reflux esophagitis in children and changes in oxidative stress, serum inflammation, vasoactive intestinal peptide and motilin. Exp. Ther. Med..

[16] Del-Toro-Sánchez C.L., Rodríguez-Félix F., Cinco-Moroyoqui F.J., Juárez J., Ruiz-Cruz S., Wong-Corral F.J., Borboa-Flores J., Castro-Enríquez D.D., Barreras-Urbina C.G., Tapia-Hernández J.A. (2021). Recovery of phytochemical from three safflower (*Carthamus tinctorius* L.) by-products: Antioxidant properties, protective effect of human erythrocytes and profile by UPLC-DAD-MS. J. Food Process. Preserv..

[17] Cho J.H., Yoon H., Shin C.M., Park Y.S., Kim N., Lee D.H. (2020). Efficacy of DA-5204 (Stillen 2X) for patients with gastroesophageal reflux disease: A randomized, double-blind, placebo-controlled pilot study. Medicine.

[18] Molina V., Valdes S., Carbajal D., Arruzazabala L., Menendez R., Mas R. (2001). Antioxidant Effect of D-002 on Gastric Mucosa of Rats with Experimentally Induced Injury. J. Med. Food.

[19] Shimoyama A.T., Santin J.R., Machado I.D., de Oliveira e Silva A.M., de Melo I.L., Mancini-Filho J., Farsky S.H. (2013). Antiulcerogenic activity of chlorogenic acid in different models of gastric ulcer. Naunyn Schmiedebergs Arch. Pharmacol..

[20] Perez Y., Oyarzabal A., Mas R., Molina V., Jimenez S. (2013). Protective effect of D-002, a mixture of beeswax alcohols, against indomethacin-induced gastric ulcers and mechanism of action. J. Nat. Med..

[21] Zamora Z., Molina V., Mas R., Ravelo Y., Perez Y., Oyarzabal A. (2014). Protective effects of D-002 on experimentally induced gastroesophageal reflux in rats. World J. Gastroenterol..

[22] Huh K., Kwon T.H., Shin U.S., Kim W.B., Ahn B.O., Oh T.Y., Kim J.A. (2003). Inhibitory effects of DA-9601 on ethanol-induced gastrohemorrhagic lesions and gastric xanthine oxidase activity in rats. J. Ethnopharmacol..

[23] Oh T.Y., Lee J.S., Ahn B.O., Cho H., Kim W.B., Kim Y.B., Surh Y.J., Cho S.W., Hahm K.B. (2001). Oxidative damages are critical in pathogenesis of reflux esophagitis: Implication of antioxidants in its treatment. Free Radic. Biol. Med..

[24] Lee J.A., Shin M.R., Kim M.J., Lee J.H., Park H.J., Roh S.S. (2021). Protective Effects of Inflammation of Curcumae Longae Rhizoma 30% EtOH Extract on Acute Reflux Esophagitis Rats. Biomed. Res. Int..

[25] Liu S., Liu H., Yan W., Zhang L., Bai N., Ho C.T. (2004). Studies on 1-O-acetylbritannilactone and its derivative, (2-O-butyloxime-3-phenyl)-propionyl-1-O-acetylbritannilactone ester. Bioorg Med. Chem. Lett..

[26] Zhang W., Dai S.M. (2012). Mechanisms involved in the therapeutic effects of Paeonia lactiflora Pallas in rheumatoid arthritis. Int. Immunopharmacol..

[27] Kim Y.S., Park H.J., Kim H., Song J., Lee D. (2019). Gastroprotective Effects of Paeonia Extract Mixture HT074 against Experimental Gastric Ulcers in Rats. Evid. Based Complement. Alternat Med..

[28] Khan A.L., Hussain J., Hamayun M., Gilani S.A., Ahmad S., Rehman G., Kim Y.H., Kang S.M., Lee I.J. (2010). Secondary metabolites from Inula britannica L. and their biological activities. Molecules.

[29] Yang L., Wang X., Hou A., Zhang J., Wang S., Man W., Yu H., Zheng S., Wang Q., Jiang H. (2021). A review of the botany, traditional uses, phytochemistry, and pharmacology of the Flos Inulae. J. Ethnopharmacol..

[30] Kim Y.S., Lee J.H., Song J., Kim H. (2020). Gastroprotective Effects of Inulae Flos on HCl/Ethanol-Induced Gastric Ulcers in Rats. Molecules.

[31] Parker S., May B., Zhang C., Zhang A.L., Lu C., Xue C.C. (2016). A Pharmacological Review of Bioactive Constituents of Paeonia lactiflora Pallas and Paeonia veitchii Lynch. Phytother. Res..

[32] Bae J.Y., Kim C.Y., Kim H.J., Park J.H., Ahn M.J. (2015). Differences in the chemical profiles and biological activities of Paeonia lactiflora and Paeonia obovata. J. Med. Food.

[33] Asai M., Kawashima D., Katagiri K., Takeuchi R., Tohnai G., Ohtsuka K. (2011). Protective effect of a molecular chaperone inducer, paeoniflorin, on the HCl- and ethanol-triggered gastric mucosal injury. Life Sci..

[34] Kim Y.S., Park H.J., Song J., Lee D., Kim H. (2018). Anti-ulcer effects of HT074 on HCl/EtOH induced gastric injury. Kor. J. Herbol..

[35] Taciak B., Bialasek M., Braniewska A., Sas Z., Sawicka P., Kiraga L., Rygiel T., Krol M. (2018). Evaluation of phenotypic and functional stability of RAW 264.7 cell line through serial passages. PLoS ONE.

[36] Elisia I., Pae H.B., Lam V., Cederberg R., Hofs E., Krystal G. (2018). Comparison of RAW264.7, human whole blood and PBMC assays to screen for immunomodulators. J. Immunol. Methods.

[37] Chun S.C., Jee S.Y., Lee S.G., Park S.J., Lee J.R., Kim S.C. (2007). Anti-inflammatory activity of the methanol extract of moutan cortex in LPS-activated Raw264.7 cells. Evid. Based Complement. Alternat Med..

[38] Hussain T., Tan B., Yin Y., Blachier F., Tossou M.C., Rahu N. (2016). Oxidative Stress and Inflammation: What Polyphenols Can Do for Us?. Oxid. Med. Cell Longev..

[39] Xu X., Li H., Hou X., Li D., He S., Wan C., Yin P., Liu M., Liu F., Xu J. (2015). Punicalagin Induces Nrf2/HO-1 Expression via Upregulation of PI3K/AKT Pathway and Inhibits LPS-Induced Oxidative Stress in RAW264.7 Macrophages. Mediators Inflamm..

[40] Jabri M.A., Tounsi H., Rtibi K., Marzouki L., Sakly M., Sebai H. (2016). Ameliorative and antioxidant effects of myrtle berry seed (*Myrtus communis*) extract during reflux-induced esophagitis in rats. Pharm. Biol..

[41] Hirschowitz B.I. (1991). A critical analysis, with appropriate controls, of gastric acid and pepsin secretion in clinical esophagitis. Gastroenterology.

[42] Omura N., Kashiwagi H., Chen G., Suzuki Y., Yano F., Aoki T. (1999). Establishment of surgically induced chronic acid reflux esophagitis in rats. Scand. J. Gastroenterol..

[43] Reimer C., Sondergaard B., Hilsted L., Bytzer P. (2009). Proton-pump inhibitor therapy induces acid-related symptoms in healthy volunteers after withdrawal of therapy. Gastroenterology.

[44] Li F., Liu H., Zhang L., Huang X., Liu Y., Li B., Xu C., Lyu J., Yin H. (2022). Effects of Gastric Acid Secretion Inhibitors for Ventilator-Associated Pneumonia. Front. Pharmacol..

[45] Peng D., Lu H., Zhu S., Zhou Z., Hu T., Chen Z., Zaika A., El-Rifai W. (2019). NRF2 antioxidant response protects against acidic bile salts-induced oxidative stress and DNA damage in esophageal cells. Cancer Lett..

[46] Itoh K., Wakabayashi N., Katoh Y., Ishii T., O’Connor T., Yamamoto M. (2003). Keap1 regulates both cytoplasmic-nuclear shuttling and degradation of Nrf2 in response to electrophiles. Genes. Cells.

[47] Kwon O.J., Choo B.K., Lee J.Y., Kim M.Y., Shin S.H., Seo B.I., Seo Y.B., Rhee M.H., Shin M.R., Kim G.N. (2016). Protective effect of Rhei Rhizoma on reflux esophagitis in rats via Nrf2-mediated inhibition of NF-kappaB signaling pathway. BMC Complement. Altern. Med..

[48] Kim S.H., Shin M.R., Lee A.R., Seo B.I., Park H.J., Roh S.S. (2020). Improvement of Inflammation through Antioxidant Pathway of Gardeniae Fructus 50% EtOH Extract (GE) from Acute Reflux Esophagitis Rats. Biomed. Res. Int..

[49] Bajpai V.K., Alam M.B., Quan K.T., Kwon K.R., Ju M.K., Choi H.J., Lee J.S., Yoon J.I., Majumder R., Rather I.A. (2017). Antioxidant efficacy and the upregulation of Nrf2-mediated HO-1 expression by (+)-lariciresinol, a lignan isolated from Rubia philippinensis, through the activation of p38. Sci. Rep..

[50] Mates J.M. (2000). Effects of antioxidant enzymes in the molecular control of reactive oxygen species toxicology. Toxicology.

[51] Pei J., Pan X., Wei G., Hua Y. (2023). Research progress of glutathione peroxidase family (GPX) in redoxidation. Front. Pharmacol..

[52] Jimenez P., Piazuelo E., Sanchez M.T., Ortego J., Soteras F., Lanas A. (2005). Free radicals and antioxidant systems in reflux esophagitis and Barrett’s esophagus. World J. Gastroenterol..

[53] Dong M., Hong T., Liu S., Zhao J., Meng Y., Mu J. (2013). Hepatoprotective effect of the flavonoid fraction isolated from the flower of Inula britannica against D-Galactosamine-induced hepatic injury. Mol. Med. Rep..

[54] Zangeneh M.M., Zangeneh A., Almasi M., Tahvilian R., Hosseini F., Moradi R. (2018). A comparative study of hepatoprotective effect of *Inula britannica* L. aqueous extract and glibenclamide in streptozotocin-induced diabetic mice. Comp. Clin. Pathol..

[55] Kim S.R., Park M.J., Lee M.K., Sung S.H., Park E.J., Kim J., Kim S.Y., Oh T.H., Markelonis G.J., Kim Y.C. (2002). Flavonoids of Inula britannica protect cultured cortical cells from necrotic cell death induced by glutamate. Free Radic. Biol. Med..

[56] Shin M.R., Lee S.H., Roh S.S. (2022). The Potential Hepatoprotective Effect of Paeoniae Radix Alba in Thioacetamide-Induced Acute Liver Injury in Rats. Evid. Based Complement. Alternat Med..

[57] Xu X., Li F., Zhang X., Li P., Zhang X., Wu Z., Li D. (2014). In vitro synergistic antioxidant activity and identification of antioxidant components from Astragalus membranaceus and Paeonia lactiflora. PLoS ONE.

